# 

**Published:** 1818

**Authors:** 


OR THE

### Archives of Veterinary Science;

CONTAINING

THE ANATOMY, PHYSIOLOGY AND PATHOLOGY

OF

### THE HORSE,

AND OTHER DOMESTIC QUADRUPEDS:

BEING THE

### OUTLINE OF A PLAN

FOR DISSEMINATING THIS MOST USEFUL AND NECESSARY BRANCH OF KNOWLEDGE;

Enabling the Gentleman, the Farmer, the Grazier, &c. not medically educated, to be his own Farrier; whereby he may practise successfully on the Diseases of his own Animals, when placed in remote Situations in different Parts of the United States, where no regular or scientific Aid can be procured.

COMPILED FROM THE LECTURES AND PRACTICE

*Of the Veterinary Colleges of London, France, Germany, Russia, and British India.*

WITH

### AN APPENDIX

CONTAINING ALL THE COLLEGE FORMULAS.

---

BY JAMES CARVER,

VETERINARY SURGEON, MASTER OF EQUITATION,

And Corresponding Member of the London Veterinary Medical Society, and the College of India.

---

*PHILADELPHIA:*

PRINTED FOR THE AUTHOR.

1818.

